# Distribution of high-risk HPV types among women in Sichuan province, China: a cross-sectional study

**DOI:** 10.1186/s12879-019-4038-8

**Published:** 2019-05-08

**Authors:** Lixia He, Junyong He

**Affiliations:** 1 Shenzhen University General Hospital, Shenzhen University Clinical Medical Academy, Guangzhou, Guangdong China; 2 Health Management Center of West China Hospital of Sichuan University, Chengdu, Sichuan China

**Keywords:** Cervical cancer screening, Cytology, HPV-DNA test, Flow cytometry, Fluorescence hybridization

## Abstract

**Background:**

Persistent infection with high-risk human papillomavirus (HR-HPV) is a major cause of cervical intraepithelial neoplasia and invasive cervical cancer. We investigated the prevalence of HR-HPV infection and distribution of viral genotypes among women in this area.

**Methods:**

Women in Sichuan older than 20 years were screened for cervical cancer between January 2015 and December 2016 using liquid-based cytology testing and a flow cytometry-fluorescence hybridization test for HPV-DNA. Frequency tables were evaluated using the chi-squared test (χ^2^).

**Results:**

Of the 17,319 women aged 20–85 years who participated in the study, Overall prevalence of HR-HPV infection was 12.6% (2178/17,319). The most prevalent viral type was HPV-52, which was present in 2.5% of individuals, followed by HPV-53 (1.6%), HPV-58 (1.5%), HPV-16 (1.1%), HPV-56 (0.9%), HPV-39 (0.8%). In HSIL, the five most common HR-HPV types were HPV52, 16, 58, 33 and 56. HPV16/18 in HSIL only makes up 25.9% of HSIL, whereas HPV31/33/45/52/58 make up 56.8%. Overall HR-HPV prevalence among women older than 65 years was 15.2%, significantly higher than the prevalence in other age groups (*P* < 0.05). Infection with dual or multiple HR-HPV types was associated with greater risk of abnormal cytology.

**Conclusion:**

Overall HR-HPV prevalence in Sichuan is as high as in large cities in China. The HR-HPV types 52, 16, 58, 33 and 56 predominated in this sample of HSIL women primarily from the banking and public sector in Sichuan. High prevalence among women older than 65 years needed pay attention to.

## Background

Cervical cancer is one of the most common cancers among women worldwide, and nearly 90% of deaths from cervical cancer occur in developing countries [1]. In China, 130,000 new cases of cervical cancer are diagnosed annually, accounting for 28% of the world total, which likely reflects the integration of the Pap smear test into the Chinese healthcare system and its widespread acceptance by women. In 2015, cervical cancer was the second most common cancer among women aged 15–45 years in China [2].

Persistent infection with high-risk HPV (HR-HPV) is a major cause of cervical intraepithelial neoplasia and invasive cervical cancer [3–5], highlighting the need for a better understanding of infection epidemiology. While HR-HPV types 16 and 18 are responsible for approximately 70% of cervical cancer cases around the world [6], and vaccines against these types are effective [7, 8] and also available in China now, many other types of HR-HPV are circulating around the world, including HPV-26, 31, 33, 35, 39, 45, 51, 52, 53, 56, 58, 59, 66, 68, and 82. These types differ greatly in their geographic distributions. For example, the five most common HR-HPV types in different regions of the world are as follows: South America, 16, 58, 18, 45, 31; North America, 16, 53, 18, 51, 31; Eastern Europe, 16, 31, 18, 66, 39; Southern Europe, 16, 66, 45, 31, 42; and Western Europe, 16, 18, 31, 35, 33 [9–12]. Even within countries, type distributions can vary widely. Within China, the five most common virus types in Zhejiang province are 11, 16, 58, 52, and 33 [13]; while the five most common in Guangdong province are 16, 52, 58, 18, and 45 [14]. Detailed understanding of HR-HPV type distribution can help inform vaccine research, development and allocation.

We are unaware of studies examining HR-HPV prevalence and genotype distribution among women in Sichuan province in China, so we undertook this large cross-sectional study.

## Methods

### Study population

The study was approved by the Hospital Ethics Committee of Sichuan University. Women from 17 cities of Sichuan province, with the highest proportions from Chengdu, Zigong, Panzhihua, Luzhou, Deyang, Mianyang, Guangyuan, Suining, Neijiang, Leshan, and Nanchong, were invited to participate in the study when they came to the Health Management Center of West China Hospital of Sichuan University for routine physical examinations between January 2015 and December 2016. Women were enrolled in the study if they were at least 20 years old, if they reported being sexually active, and if they provided written informed consent. Women were excluded from the study if they were pregnant or if they had a history of immunodeficiency disease, cervical cone or uterus resection, or acute genital inflammation. Pregnancy status and immunodeficiency history were assessed based on interviews and medical records.

### Cervical specimen collection

Cervical cells were obtained from the cervical canal using two plastic brushes. One brush was placed into a 10-mL vial containing BD SurePath preservative fluid (TriPath Imaging, Burlington, NC, USA) for later cytology analysis. The other brush was placed into a 2-mL vial of preservation solution (Tellgen Life Science, Shanghai, China) for subsequent HPV-DNA testing. Samples for cytology were stored at room temperature for no more than 4 weeks, or at 4 °C for no more than 6 months. Samples for HPV-DNA tests were stored at room temperature for no more than 2 days, or at 2–8 °C for no more than 2 months.

### Liquid-based cytology

Liquid-based cytology analysis was performed in the Pathology Department of West China Hospital of Sichuan University. Cytology specimens were evaluated independently by two experienced cytopathologists according to the 2001 Bethesda System [15]. Cytopathologists were blinded to the results of other assays. If the two experts came to different diagnoses, the cervical samples were reviewed again and a consensus diagnosis was obtained. The following diagnoses were possible: negative for intraepithelial lesion or malignancy (NILM), atypical squamous cells of undetermined significance (ASC-US), atypical squamous cells with the possibility of high-grade squamous intraepithelial lesion (ASC-H), low-grade squamous intraepithelial lesion (LSIL), high-grade squamous intraepithelial lesion (HSIL), squamous cervical cancer (SCC), atypical glandular cells (AGC), or adenocarcinoma in situ (AIS).

### HPV-DNA extraction and genotyping

DNA was extracted using a commercial DNA Mini Kit (Tellgen Life Science) according to the manufacturer’s instructions. DNA concentrations were determined using a NanoDrop1000 spectrophotometer, and samples were frozen at − 20 °C until analysis.

HPV-DNA was detected using a flow cytometry-fluorescence hybridization technology that has been approved by the Chinese Food and Drug Administration for clinical use and has shown good diagnostic performance [16, 17]. This assay uses nine sense primers (BSGP5+, 200 nM) and three antisense primers (5′-biotinylated-BSGP6+, 400 nM) to amplify a ~ 150-bp fragment of the viral L1 open reading frame. At the same time, the assay contains primers MS3 and MS10 (300 nM) to amplify the β-globin gene as an internal control of DNA integrity. This assay can identify the following HR-HPV genotypes: 16, 18, 26, 31, 33, 35, 39, 45, 51, 52, 53, 56, 58, 59, 66, 68, and 82. (It can also identify 10 low-risk HPV types.)

Oligonucleotide probes against each of the 17 HR-HPV types and 10 low-risk HPV types were synthesized with an amino group at the 5′ end. All PCR procedures were performed according to the manufacturer’s recommendations. The mixture of PCR products and conjugate was incubated for 20 min at room temperature with shaking. After three washes in blocking solution, the beads were analyzed in a Luminex 200 reader (Luminex Corporation, TX, USA). This reader uses one laser to identify the bead type based on bead color, and a second laser to quantify reporter fluorescence on the bead. Median fluorescence intensity (MFI) for ≥100 beads was measured [18–20].

MFI was used to stratify participants as having low or high HPV load based on calibration using plasmid standards from the World Health Organization Panel II [21] with 10^2^ and 10^3^ copies of 16 HPV types per 100 ng of human placental DNA. MFIs obtained with 10^3^ HPV genome copies of the respective HPV types per 100 ng of human placental DNA were used as cut-off values for classifying women as having low or high load of the respective HPV type.

### Statistical analysis

All analyses were performed using SPSS 19.0 for Windows (IBM, Armonk, NY). Prevalence of HR-HPV infection, genotype distribution, and single and multiple HPV infections were analyzed. Prevalence of HR-HPV(n) = number of positive HR-HPV(n)/included objects. The significance of frequency distributions was evaluated using the chi-squared test (χ^2^), and the associated 95% confidence intervals (CIs) were calculated using a binomial distribution analysis. For all analyses, *P* < 0.05 was considered statistically significant.

## Results

### Prevalence of HR-HPV infection

A total of 17,319 women aged 20–85 years were included in this study. Their average age was 43.56 ± 10.22 years and 80% were teachers, policewomen, government workers, bank workers, civil servants, or retirees; the type of employment and working/retired status were unknown for the remaining 20%. All 27 HPV types that our HPV-DNA assay can detect were detected in this population, including the 17 HR-HPV types 16, 18, 26, 31, 33, 35, 39, 45, 51, 52, 53, 56, 58, 59, 66, 68, and 82. The remaining 10 types belonged to low-risk HPV, which will not be discussed here.

Overall prevalence of HR-HPV infection was 12.6% (2178/17,319). The most prevalent type was HPV-52, which was detected in 424 women, corresponding to an infection prevalence of 2.5%. The next most prevalent types were 53 (1.6%), 58 (1.5%), 16 (1.1%), 56 (0.9%), 39 (0.8%), 59 (0.7%), 66 (0.7%), 18 (0.6%), and 51 (0.6%) (Table 1). We divided these HR-HPV into three groups based on the HPV vaccine, the prevalence of HPV16/18 is 1.7% (300/17319), HPV31/33/45/52/58 is 4.71% (816/17319), HPV:26/35/39/51/53/56/59/66/68/82 is 6.1% (1062/17319).

**Table 1:** Distribution of HR-HPV infections among women of different age^a^

HPV type	20-29y(%) *n* = 1361	30-39y(%) *n* = 4923	40-49y(%) *n* = 6227	50-59y(%) *n* = 3478	60-65y(%) *n* = 936	>65 y(%) *n* = 394	Total *n* = 17,319)
16	19 (1.4)	57 (1.2)	56 (0.9)	38 (1.1)	15 (1.6)	4 (1.0)	189 (1.1)
18	12 (0.9)	25 (0.5)	40 (0.6)	17 (0.5)	11 (1.2)	6 (1.5)	111 (0.6)
26	2 (0.2)	0 (0.0)	2 (0.0)	2 (0.1)	2 (0.2)	1 (0.3)	9 (0.1)
31	4 (0.3)	7 (0.1)	13 (0.2)	5 (0.1)	2 (0.2)	0 (0.0)	31 (0.2)
33	2 (0.2)	21 (0.4)	24 (0.4)	19 (0.6)	9 (1.0)	5 (1.3)	80 (0.5)
35	5 (0.4)	15 (0.3)	23 (0.4)	13 (0.4)	5 (0.5)	2 (0.5)	63 (0.4)
39	10 (0.7)	46 (1.0)	39 (0.6)	33 (1.0)	11 (1.2)	4 (1.0)	143 (0.8)
45	2 (0.2)	9 (0.1)	9 (0.1)	6 (0.2)	1 (0.1)	3 (0.8)	27 (0.2)
51	11 (0.8)	39 (0.8)	37 (0.6)	16 (0.5)	4 (0.4)	2 (0.5)	109 (0.6)
52	39 (2.9)	120 (2.4)	143 (2.3)	85 (2.4)	30 (3.2)	7 (1.8)	424 (2.5)
53	22 (1.6)	69 (1.4)	101 (1.6)	62 (1.8)	15 (1.6)	12 (3.1)	281 (1.6)
56	8 (0.6)	36 (0.7)	49 (0.8)	30 (0.9)	13 (1.4)	11 (2.8)	147 (0.9)
58	15 (1.1)	63 (1.3)	83 (1.3)	51 (1.5)	28 (3.0)	14 (3.6)	254 (1.5)
59	11 (0.8)	25 (0.5)	42 (0.7)	28 (0.8)	10 (1.1)	7 (1.8)	123 (0.7)
66	11 (0.8)	28 (0.6)	47 (0.8)	19 (0.6)	7 (0.8)	6 (1.5)	118 (0.7)
68	6 (0.4)	6 (0.1)	7 (0.1)	8 (0.2)	1 (0.1)	4 (1.0)	32 (0.2)
82	4 (0.3)	8 (0.2)	13 (0.2)	8 (0.2)	4 (0.4)	0 (0.0)	37 (0.2)

^a^Values in the age columns refer to n (column%)

**Fig. 1:**
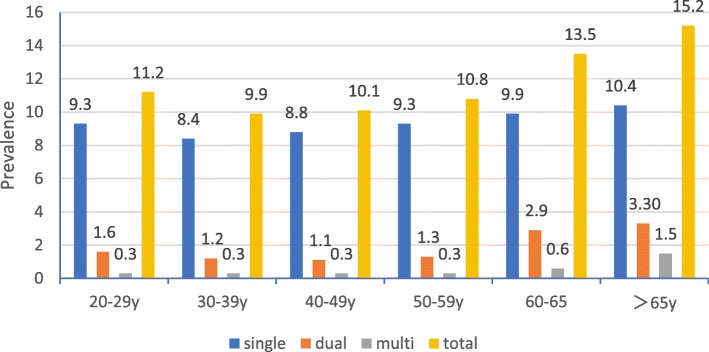
Age-specific HR-HPV prevalence among women in Sichuan

**Fig. 2:**
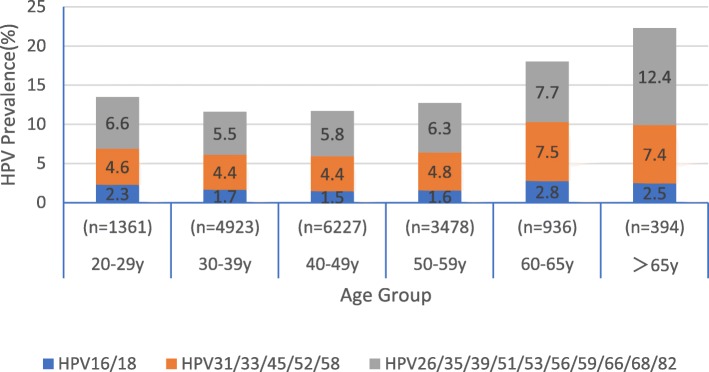
prevalence of three group HR-HPV infections among women of different age

The prevalence of HR-HPV infection was highest among women older than 65 years 15.2%, followed by a prevalence of 13.5% among women aged 60–65 and 11.2% among women aged 20–29 (Fig. 1). These percentages differed significantly (*P* < 0.05). Across all age groups, the 10 most frequent HR-HPV types were 52, 53, 58, 16, 18, 59, 66, 51, 39, 56 (Table 1), although the relative order of frequencies varied with age group. In different age groups, the prevalence of HPV:26/35/39/51/53/56/59/66/68/82 is the highest, followed by HPV31/33/45/52/58, and then HPV16/18 (Fig. 2).

### Prevalence of infection with single, dual or multiple HR-HPV types among women of different age

The prevalence of infection with a single HR-HPV type was 10.4% among women older than 65, followed by 9.9% among women aged 60–65, and 9.3% among women aged 20–29 or 50–59 (Fig. 1). The highest prevalence of dual HR-HPV infection was 3.3% among women older than 65, 2.9% among women aged 60–65, and 1.6% among women aged 20–29. The highest prevalence of multiple HR-HPV infection was 1.5% among women older than 65, followed by 0.6% among women aged 60–65, and lower rates among women aged 20–29 or 50–59.

### Relationship between HR-HPV infection and cytology results

As shown in Table 2, of the 17,319 samples, 97.2% (16,832/17319) were diagnosed as NILM and 2.8% (487/17319) as abnormal cytology, comprising 1.1% (192/17,319) with ASC-US, 1.0% (172/17,319) with LSIL, 0.5% (81/17,319) with HSIL (including HSIL and ASC-H) and 0.2% (42/17,319) with AGC. The prevalence of HR-HPV infection among women diagnosed with NILM was 11.1% (1868/16,832), significantly lower than the prevalence among women diagnosed with abnormal cytology (63.7%, 310/487, *P* < 0.05).

**Table 2:** Distribution of HR-HPV infections among women of different cytology

HPV type	NILM,% *n* = 16,832	ASCUS,% *n* = 192	LSIL,% *n* = 172	HSIL,% *n* = 81	AGC,% *n* = 42	Total (*n* = 17,319)
16	154 (81.5)	6 (3.2)	11 (5.8)	18 (9.5)	0	189 (100%)
18	93 (83.8)	6 (5.4)	8 (7.2)	3 (2.7)	1 (0.9)	111 (100%)
26	8 (88.9)	0	0	1 (11.1)	0	9 (100%)
31	23 (74.2)	2 (6.5)	3 (9.7)	3 (9.7)	0	31 (100%)
33	56 (70.0)	7 (8.8)	9 (11.3)	8 (10.0)	0	80 (100%)
35	52 (82.5)	3 (4.8)	6 (9.5)	2 (3.2)	0	63 (100%)
39	136 (95.1)	0 (0)	6 (4.2)	1 (0.7)	0	143 (100%)
45	25 (92.6)	0 (0)	1 (3.7)	1 (3.7)	0	27 (100%)
51	96 (88.1)	4 (3.7)	7 (6.4)	2 (1.8)	0	109 (100%)
52	351 (82.8)	24 (5.7)	28 (6.6)	20 (4.7)	1 (0.2)	424 (100%)
53	259 (92.2)	5 (1.8)	14 (5.0)	3 (1.1)	0	281 (100%)
56	122 (83.0)	4 (2.7)	17 (11.6)	4 (2.7)	0	147 (100%)
58	211 (83.1)	12 (4.7)	17 (6.7)	14 (5.5)	0	254 (100%)
59	114 (92.7)	3 (2.4)	4 (3.3)	2 (1.6)	0	123 (100%)
66	108 (91.5)	1 (0.8)	8 (6.8)	1 (0.9)	0	118 (100%)
68	27 (84.4)	0 (0)	4 (12.5)	1 (3.1)	0	32 (100%)
82	33 (89.2)	2 (5.4)	1 (2.7)	1 (2.7)	0	37 (100%)

Values in the cytology rows refer to n (row%)

Except 1 case of HSIL caused by HPV26 combined with HPV16 infection, 10% HPV33 infection leads to HSIL ranked NO.1, followed by HPV31(9.7%), HPV16(9.5%), HPV58(5.5%), HPV52(4.7%), HPV45(3.7%).

The prevalence among women diagnosed with ASCUS was 31.8% (61/192); among women with LSIL, 61.6% (106/172); and among women with HSIL, 77.8% (63/81). These percentages were significantly higher than the percentages among women diagnosed with NILM (9.5%, 1596/16,832) or AGC (2.4%, 1/42). No relationship was observed between AGC and HR-HPV infection. Different HR-HPV types predominated in different cytology groups, the five most frequent types among women with HSIL were 52, 16, 58, 33 and 56. In contrast, the five most frequent types among women with LSIL were52, 56, 58,53 and 16. In the form that HPV16/18 only makes up 25.9% (21/81) of HSIL, whereas HPV31/33/45/52/58 make up 56.8% (Table 3).

**Table 3:** Prevalence of three HR-HPV groups according to different cytology results

HPV types	NILM	ASCUS	LSIL	HSIL	AGC	Total
	*n* = 16,832	*n* = 192	*n* = 172	*n* = 81	*n* = 42	(*n* = 17,319)
16/18	247 (1.5)	12 (6.3)	19 (11.1)	21 (25.9)	1 (2.4)	300 (1.7)
31/33/45/52/58	666 (4.0)	45 (23.4)	58 (33.7)	46 (56.8)	1 (2.4)	816 (4.7)
26/35/39/51/53/56/59/66/68/82	1062 (6.3)	22 (11.5)	67 (39.0)	18 (22.2)	0 (0.0)	1062 (6.1)

Values in the cytology columns refer to n (column%)

### Relationship between HR-HPV infection number and cytology results

Compared with HR-HPV prevalence among the 16,832 cytologically normal participants, OR (95% CI) was 3.6(2.6–5.2) in the ASCUS group, 13.1(9.4–18.3) in the LSIL group, and 27.7(16.0–48.0) in the HSIL group in the case of individuals with single HR-HPV infection. The corresponding results in the case of individuals with dual HR-HPV infection were 11.9(7.1–19.9, 24.92(14.7–42.4) and 76.95 (38.6–153.4). In the case of individuals with multiple HR-HPV infection, number of ASC was zero, OR (95% CI) was 46.2 (21.7–98.3) in the LSIL group, and 37.62 (8.5–166.9) in the HSIL group. The wide 95%CIs in the HSIL group reflect the small number of such women in our sample. These results indicated that the severity of abnormal cytology in an infected individual increased with the number of HR-HPV types (Table 4).

**Table 4:** Relationship between HR-HPV infection number and cytology results

No. HR-HPV	Cytology diagnosis, n (column%)				
	NILM	ASCUS	LSIL	HSIL	AGC
cases detected*	(*n* = 16,832)	(*n* = 192)	(*n* = 172)	(*n* = 81)	(*n* = 42)
Negative	15,236 (90.5)	131 (68.2)	66 (38.4)	18 (22.2)	41 (97.6)
Single	1375 (8.2)	43 (22.4)	78 (45.4)	45 (55.6)	0
OR (95%CI)	1	3.64 (2.6–5.2)*	13.10 (9.4–18.3)*	27.70 (16.0–48.0)*	
Dual	176 (1.1)	18 (9.4)	19 (11.1)	16 (19.8)	1 (2.4)
OR (95%CI)	1	11.90 (7.1–19.9)*	24.92 (14.7–42.4)*	76.95 (38.6–153.4)*	2.11 (0.3–15.4)
Multi	45 (0.3)	0 (0)	9 (5.2)	2 (2.5)	0
OR (95%CI)	1		46.17 (21.7–98.3)*	37.62 (8.5–166.9)*	

ORs were calculated relative to the Negative group. Trend test: * *p* < 0.05

## Discussion

This is the first large study, to our knowledge, of HR-HPV prevalence among women in Sichuan province of China, and of the relationship between HR-HPV infection and abnormal cytology. In our sample of 17,319 women, primarily from the banking and public sectors, the overall rate of HR-HPV infection was 12.6%, and the five most common types of HR-HPV were 52, 53, 58, 16 and 56. Our overall frequency of HR-HPV infection (12.6%) is similar to frequencies reported for the large Chinese cities Tianjin (12.5%) [22], Guangdong (7.3%) [23], and Beijing (7.0%) [24], but it is lower than the frequency reported for Shanghai (31.8%) [25], Fujian (18.7%) [26], Yunnan (18.4%) [27], and Qingdao (32.2%) [28]. Higher prevalence in some other studies may reflect that we recruited asymptomatic women who came to our center for routine physical check-ups rather than hospital patients, who may show higher HR-HPV prevalence. Discrepancies between our study and others may also reflect geographic or socioeconomic differences in virus prevalence. Further work should clarify to what extent these results are influenced by differences in demographics of the study samples and in virus testing methods.

In our study, the HR-HPV infection rates of the group aged over 60 is much higher, compared with other groups, which is similar to results of a study on women in Shenzhen, China [29]. A community-based survey of 10,602 Taiwan women showed that HPV prevalence was highest in women aged 60–65 (20.1%) [30]. A study consisting of 10,442 Fujian women showed that HPV prevalence showed a bi-modal trend with age [26]. A study of Shanghai women visiting a cervical disease diagnosis and treatment center showed an HR-HPV prevalence of 31.5% among women aged ≥65, which was higher than in women aged 35–54 [25]. We speculate that because of the discomfort and embarrassment of gynecological examination, the participation rate of cervical cancer screening in the elderly is low, and most of the participants participate in the screening because of the discomfort symptoms of reproductive tract, so the positive rate is high. However, these results contrast with reports of declining infection rates with age in studies from Colombia, Japan [31, 32]. These discrepancies may reflect ethnic differences.

The most common HR-HPV types in Sichuan (52, 53, 58, 16, 56) appear to differ from those in other Chinese cities, such as Shanghai (52, 16, 18, 31, 18) [25], Fujian (16, 52, 58, 18, 53) [26], Tianjin (16, 18, 58, 66) [22], Guangdong (52, 58, 16, 18) [23] and Hunan (16, 52, 58, 18) [33].

Despite this geographic variation, this difference may be related to the included population. Some studies were patient specimens collected in the department of obstetrics and gynecology, while some were only targeted at the general population, and some were a combination of the two groups. The other potential reason for this discrepancy with other studies is due to the sample size, while our study included 17,319 women. It is worth mentioning that despite the high positive rate of HPV53, the cytological results of over 90% HPV53 positive women were NILM.

We further analyzed the most common HPV genotype in HSIL. In this study, HPV types 52, 16, 58, 33 and 56 were the five most common HR-HPV types in HSIL. The first three most common HR-HPV types consisted with other studies in many cities in China. HPV-52 and HPV-58 are also very common in Hong Kong and Taiwan [34, 35]. However, in our study, HPV16/18 only makes up 25.93% of HSIL, whereas HPV31/33/45/52/58 make up 56.8%. when we look at HPV prevalence in cancer, a meta-analysis showed that in cell-based detection, HPV16/58/18/52/33 were the five most frequently detected HR-HPV type in ICC in many studies, HPV31, 33, 52, and 58 were more frequent in CIN3 in comparison with cervicitis/normal samples, but less common in ICC [36]. The possible reason need further investigated.

Furthermore, we found that HR-HPV prevalence increased with severity of abnormal cytology, consistent with previous studies [22, 25–27]. This likely reflects the well-established association between HR-HPV and risk of cervical cancer. We further found that the presence of two or more HR-HPV types was associated with greater likelihood of abnormal cytology, also consistent with previous work [23, 37, 38]. This may be a virus load effect, though it may also reflect interaction between particular virus types. Further research should examine why multiple HR-HPV infection leads to greater risk of abnormal cytology.

Our epidemiological results on HR-HPV types in Sichuan province, with a female population of more than 40 million, may have immediate implications for Chinese public health practices. In 2017, the Chinese government approved vaccines effective against HPV-16 and HPV-18, which together cause approximately 70% of cervical cancers around the world [6]. However, in this study, HPV16/18 only makes up 25.9% of HSIL, whereas HPV31/33/45/52/58 make up 56.8%. Thus, the results indicate that bivalent HPV vaccine may fail to protect a substantial proportion of women from cervical intraepithelial neoplastic lesions and cervical cancer. This highlights the need for 9-valent human papillomavirus vaccine to cover this large group of vulnerable women.

Despite the large size and careful correlation between HR-HPV positivity and abnormal.

cytology, our findings should be interpreted with caution in light of limitation. Our study involved primarily healthy, working women in urban, so the results may not be generalizable to remote rural female populations. However, a multicenter population-based study from Beijing, Shanghai, Shanxi, Henan and Xinjiang representing both urban and rural areas indicated that the HPV prevalence in CIN women from rural and urban have no significant difference [39]. In this study, we only analyzed the relationship between HPV and cytology, not histology. Cytology is also not as good as histology, as cytology is an imperfect test, we will further explore the relationship between HPV and histology in the next study.

Despite these limitations, our epidemiological study of HR-HPV in Sichuan province suggests that the substantial prevalence reported in several large Chinese cities may be true for a large section of the country. We also found that infection with multiple HPV types was associated with greater risk of abnormal cytology, which may help guide future studies into how HPV infection leads to cervical cancer.

## Conclusion

The overall HR-HPV prevalence in Sichuan may be as high as in large cities in China. HR-HPV types 52, 53, 58, 16, and 56 predominate among infected women in Sichuan. Infection with two or more HR-HPV types may increase their risk of abnormal cytology. The high HR-HPV prevalence among the elder women deserve further attention.

## Abbreviations

AGC: Atypical glandular cells
AIS: Adenocarcinoma in situ
ASC-H: Atypical squamous cells with the possibility of high-grade squamous intraepithelial lesion
ASC-US: Atypical squamous cells of undetermined significance
DNA: Deoxyribonucleic acid
HR-HPV: High-risk human papilloma virus
HSIL: High-grade squamous intraepithelial lesion
ICC: Invasive cervical cancer
LSIL: Low-grade squamous intraepithelial lesion
NILM: Negative for intraepithelial lesion or malignancy
PCR: Polymerase chain reaction
SCC: Squamous cervical cancer
STD: Sexually transmitted disease
